# Stochastic Integration H_∞_ Filter for Rapid Transfer Alignment of INS

**DOI:** 10.3390/s17112670

**Published:** 2017-11-18

**Authors:** Dapeng Zhou, Lei Guo

**Affiliations:** 1 School of Instrumentation Science and Opto-Electronics Engineering, Beihang University, Beijing 100191, China; 2 Science and Technology on Aircraft Control Laboratory, Beihang University, Beijing 100191, China; lguo@buaa.edu.cn; 3 School of Automation Science and Electrical Engineering, Beihang University, Beijing 100191, China

**Keywords:** inertial navigation system, rapid transfer alignment, stochastic integration H_∞_ filter

## Abstract

The performance of an inertial navigation system (INS) operated on a moving base greatly depends on the accuracy of rapid transfer alignment (RTA). However, in practice, the coexistence of large initial attitude errors and uncertain observation noise statistics poses a great challenge for the estimation accuracy of misalignment angles. This study aims to develop a novel robust nonlinear filter, namely the stochastic integration H${}_{\infty }$ filter (SIH${}_{\infty }$F) for improving both the accuracy and robustness of RTA. In this new nonlinear H${}_{\infty }$ filter, the stochastic spherical-radial integration rule is incorporated with the framework of the derivative-free H${}_{\infty }$ filter for the first time, and the resulting SIH${}_{\infty }$F simultaneously attenuates the negative effect in estimations caused by significant nonlinearity and large uncertainty. Comparisons between the SIH${}_{\infty }$F and previously well-known methodologies are carried out by means of numerical simulation and a van test. The results demonstrate that the newly-proposed method outperforms the cubature H${}_{\infty }$ filter. Moreover, the SIH${}_{\infty }$F inherits the benefit of the traditional stochastic integration filter, but with more robustness in the presence of uncertainty.

## 1. Introduction

The inertial navigation system (INS) is an entirely autonomous navigation system, in the sense that it precisely provides the navigation parameters for the carrier without the aid of a signal from an external device. As is well known, the performance of INS operated on a moving base is heavily influenced by the accuracy of transfer alignment, which is an important research interest of modern navigation techniques. In particular, the alignment issue between a main INS (MINS) and a slave INS (SINS) has drawn extensive attention, partially due to its widespread application in diverse carriers. For the MINS/SINS system, the unknown parameters of SINS are commonly initialized by MINS at the very beginning. The inevitable initial misalignments will lead to poor accuracy of the follow-on navigation operation, and thus, the alignment stage of SINS is necessitated. In [1], the rapid transfer alignment (RTA) on the basis of the “attitude plus velocity” matching scheme was originally proposed to estimate the attitude errors between two INSs. Then, the misalignment angles of SINS with respect to the local navigation frame can be indirectly determined. Compared with traditional transfer alignment, the RTA has superiorities in both model observability and convergence rate, and it has been widely applied for both military and civilian purpose [1,2,3].

The central concerns of RTA are the estimation accuracy and convergence rate, both of which are intimately related to the performance of the filtering algorithm. For the RTA with the linearized model and Gaussian noise, the capacity of the Kalman filter (KF) that provides optimal estimation has been authoritatively verified through numerical simulation and field tests [1]. However, the INS sometimes has to be initiated in a complicated environment, and higher demanding standards are put forward to the filtering algorithm. First, for some quick response missions, the initial misalignment angles of INS may be notably large, and significant nonlinearity will be brought into the model description of RTA [2]. That is, the nonlinear character of the filtering algorithm should be deliberately considered. In addition, the precision of observations severely influences the performance of RTA. Nevertheless, in practice, the phenomenon of uncertain observations frequently occurs due to the existence of multiple disturbances, including outliers, the time delay of data transmission, the coupling effect between the lever arm and deformation, etc. [4,5]. The suppression of negative effects induced by these disturbances is of crucial importance to the improvement of the accuracy and stability of RTA. For this purpose, accurate modeling methods have been developed as intuitive solutions for the compensation of these disturbances [6,7,8]. Yet, the complexity of the model will be severely increased, and the un-modeled errors will destroy the veracity of model. Another way is to take these disturbances as uncertain interfering input of the model, and against the negative effect by means of the robust filtering algorithm. On the premise of the bounded energy of noise, one effective method is the H${}_{\infty }$ filter, which intends to minimize the downside on estimation results in the worst case of the un-modeled disturbance and uncertainty. Contrary to KF, the H${}_{\infty }$ filter requires neither statistical noise properties, nor the exact system model and provides robust and accurate estimations, while the uncertain parameters exist in the model description [9].

Many solutions, as exemplified by the Krein space approach and the game theory approach, have been developed for the linear H${}_{\infty }$ problem [9,10,11,12]. Afterward, these solutions are naturally adopted for nonlinear systems with the utilization of the first-order linearization method; to be more precise, the extended H${}_{\infty }$ filter (EH${}_{\infty }$F) [13,14]. However, the EH${}_{\infty }$F inherits the drawbacks of the extended Kalman filter, i.e., the cumbersome computation of Jacobians and the deterioration of estimation performance in the presence of significant nonlinearity. Recent decades have witnessed the development of sample-point filters on the basis of deterministic sampling quadrature methods (as exemplified by the unscented transform, the cubature integration rule, the Gauss–Hermite quadrature rule, etc.), which either approximate the probability distribution function representing state estimates by a set of sample-points or approximate the nonlinear functions using polynomial expansions [15,16,17,18]. The emergence of these quadrature methods facilitates the arising of the derivative-free H${}_{\infty }$ filter (DFH${}_{\infty }$F), such as the unscented H${}_{\infty }$ filter, cubature H${}_{\infty }$ filter (CH${}_{\infty }$F) and sparse-grid quadrature H${}_{\infty }$ filter [19,20,21]. Compared with EH${}_{\infty }$F, the DFH${}_{\infty }$F releases the restriction of computing Jacobians and achieves high accuracy, as the posterior estimations are accurately approximated to certain order. However, the approximations given by deterministic sampling quadrature methods cannot eliminate systematic errors that exist in the solution of DFH${}_{\infty }$F [15]. That is, the estimation accuracy will be degraded while significant nonlinearity exists in the model description. Moreover, the boundedness and convergence of estimation error cannot be guaranteed due to the local validity of these quadrature methods. To overcome these disadvantages, as of late, a novel filter, named the stochastic integration filter (SIF), was proposed to provide asymptotically exact estimation for nonlinear systems [22,23,24]. The core of SIF is the stochastic spherical-radial integration rule (SSRIR), which approximates the probability distribution function of posterior estimation by a set of randomly-chosen sample-points [25]. Contrary to the deterministic sampling quadrature methods, the SSRIR can theoretically eliminate the systematic error with increasing iterations. Therefore, it can be reasonably concluded that the incorporation of SSRIR with the framework of DFH${}_{\infty }$F will result in a filter with high accuracy and strong robustness, but there were few published literature works focused on this study. This paper is devoted to developing a stochastic integration H${}_{\infty }$ filter (SIH${}_{\infty }$F) to address the RTA issue with the coexistence of significant nonlinearity and uncertain observation noise statistics. For the first time, the SSRIR is combined with the framework of DFH${}_{\infty }$F, and the resulting SIH${}_{\infty }$F achieves estimation with both accuracy and robustness. Numerical simulation and the van test are separately executed for the validation of the proposed method. The results demonstrate that the SIH${}_{\infty }$F has better performance than the previous, well-known CH${}_{\infty }$F and SIF.

The rest of this paper is organized as follows: The nonlinear model of RTA is briefly introduced in Section 2. Section 3 firstly represents the preliminaries of the H${}_{\infty }$ technique and the EH${}_{\infty }$F. Then, the derivation of SIH${}_{\infty }$F is described in detail. Numerical simulation is executed in Section 4, in order to demonstrate the superiorities of SIH${}_{\infty }$F compared with the CH${}_{\infty }$F and SIF. In Section 5, the validity of the proposed method is further verified through a van test. The conclusions are given in Section 6.

## 2. System Modeling of Rapid Transfer Alignment

The aim of system modeling is to describe the error propagation principle of RTA. In this section, the process model and observation model of RTA with the nonlinear characteristic are briefly introduced. The system modeling lays the foundation for the development of the filtering algorithm in the following section.

**Figure 1:**
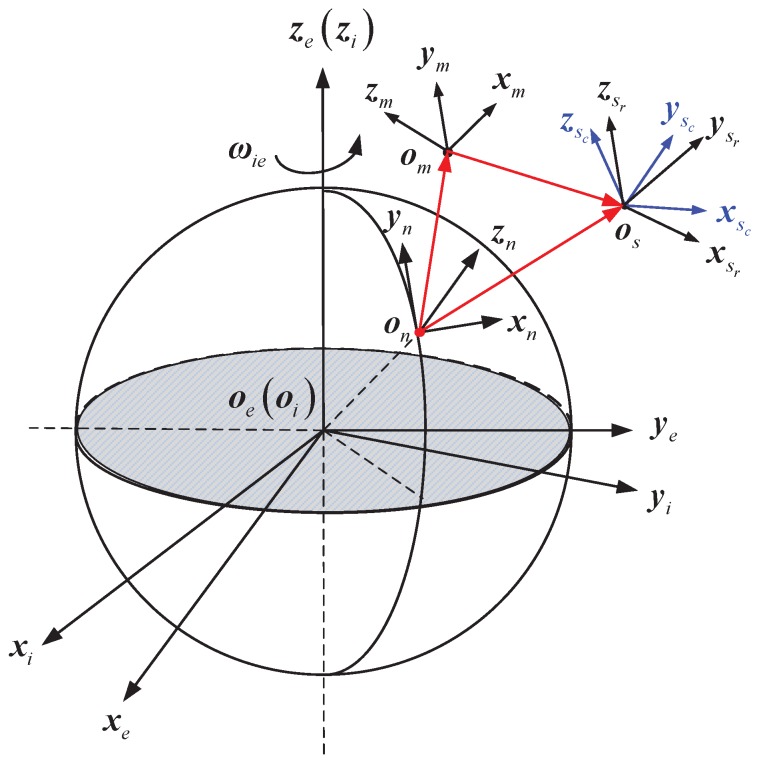
Definitions of the coordinate systems.

The definitions of coordinate systems used in system modeling are shown in Figure 1, where *i*-frame denotes the inertial frame, *e*-frame denotes the Earth-centered Earth-fixed frame, *n*-frame denotes the local navigation frame, *m*-frame denotes the body frame of MINS, $s_{r}$-frame denotes the body frame of SINS and $s_{c}$-frame denotes the calculated body frame of SINS. Note that all of these coordinated systems are right handed and Cartesian; the detailed specifications of these coordinate systems can be found in [2].

**Figure 2:**
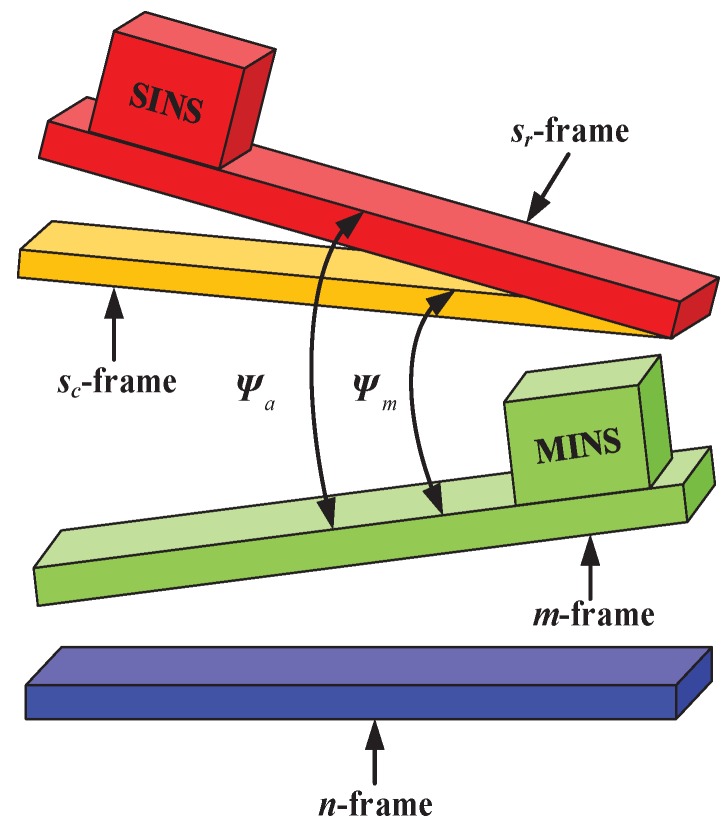
Sketch map of misalignment angles between the main INS (MINS) and slave INS (SINS).

In this paper, the two INSs are assumed to be directly installed on the carrier, and the sketch map of misalignment angles between the MINS and SINS is represented in Figure 2, where $\psi_{m}$ represents the misalignment angles between the *m*-frame and the $s_{c}$-frame and $\psi_{a}$ represents the misalignment angles between the *m*-frame and the $s_{r}$-frame. The eventual purpose of RTA is to determine the attitude transformation matrix from $s_{r}$-frame to *n*-frame. Since the errors of MINS have been well compensated by the external aid of the device, the attitude parameters given by MINS can be viewed as reliable. In other words, the goal of RTA can be indirectly achieved by estimating the attitude errors between the two INSs, i.e., $\psi_{a}$. However, due to the inherent accumulative errors of sensors, only the inertial data sensed by SINS projected in the $s_{c}$-frame imply the misalignment information. Therefore, the description of error propagation is the prerequisite of RTA.

Generally, the process model of RTA includes the attitude error equation, velocity error equation, dynamic models of inertial sensors and actual physical misalignment angles. The attitude error equation mainly describes the function relationship between $\psi_{m}$ and $\psi_{a}$. Assuming that large misalignment angles exist in all three axes, the attitude error equation of RTA is given by [2]:

(1) $${\dot{\psi }}_{m}=\Xi \left[\left(I_{3\times 3}-C_{m}^{s_{c}}C_{s_{r}}^{m}\right)\omega_{ns_{c}}^{s_{c}}+C_{m}^{s_{c}}C_{s_{r}}^{m}\varepsilon^{s_{r}}\right]+w_{m}$$

where Ξ is defined as:

(2) $$\Xi =\begin{array}{c} \left(\begin{array}{ccc} \cos\psi_{my} & 0 & \sin\psi_{my}\\ \sin\psi_{my}\tan\psi_{mx} & 1 & -\cos\psi_{my}\tan\psi_{mx}\\ -\sin\psi_{my}/\cos\psi_{mx} & 0 & \cos\psi_{my}/\cos\psi_{mx} \end{array}\right) \end{array}$$

and $\psi_{m,i}$, (i=x,y,z) denotes the measurable misalignment angle in the corresponding axis; $I_{3\times 3}$ is the unit matrix; $C_{m}^{s_{c}}$ denotes the attitude transformation matrix from the *m*-frame to the $s_{c}$-frame; $C_{s_{r}}^{m}$ represents the attitude transformation matrix from the $s_{r}$-frame to the *m*-frame; $\omega_{ns_{c}}^{s_{c}}$ is the angular velocity of the $s_{c}$-frame relative to the *n*-frame projected in the $s_{c}$-frame; $\varepsilon^{s_{r}}$ is the constant drift of the gyroscope with dynamic model ${\dot{\varepsilon }}^{s_{r}}=0_{3\times 1}$; $w_{m}$ is the noise term of the attitude error equation.

On the basis of the velocity solution given by the strapdown algorithm, the velocity error equation can be described as [2]:

(3) $$\delta {\dot{V}}^{n}=C_{s_{c}}^{n}\left(I_{3\times 3}-C_{m}^{s_{c}}C_{s_{r}}^{m}\right)f_{is_{r}}^{s_{r}}-\left(2\omega_{ie}^{n\times }+\omega_{en}^{n\times }\right)\delta V^{n}+C_{s_{r}}^{n}\nabla^{s_{r}}+w_{v}$$

where $\delta V^{n}$ is the velocity error vector projected in the *n*-frame; $\omega_{ie}^{n}$ is the angular velocity of the *e*-frame relative to the *i*-frame projected in the *n*-frame; $f_{is_{r}}^{s_{r}}$ is the specific force sensed by SINS projected in the $s_{r}$-frame; $\omega_{en}^{n}$ is the angular velocity of the *n*-frame relative to the *e*-frame projected in the *n*-frame; $C_{m}^{n}$ is the attitude transformation matrix from the *m*-frame to the *n*-frame, and it is commonly provided by MINS in real time; $\nabla^{s_{r}}$ is the constant bias of the accelerometer with dynamic model ${\dot{\nabla }}^{s_{r}}=0_{3\times 1}$; $w_{v}$ is the noise term of the velocity error equation. Note, the derivation of Equation (3) assumes that the acceleration induced by the lever arm effect has been well compensated.

Due to the impact of vibration and flexure, the modeling of $\psi_{a}$ makes a compromise between the complexity and accuracy, that is [1]:

(4) $${\dot{\psi }}_{a}=\eta_{a}$$

where $\eta_{a}$ is the noise term with covariance $Q_{a}$.

Choosing the state vector as $x={\left[\begin{array}{ccccc} \psi_{m} & \delta V^{n} & \varepsilon^{s_{r}} & \nabla^{s_{r}} & \psi_{a} \end{array}\right]}^{T}$, the process model of RTA can be represented as follows:

(5) $$\left\{\begin{array}{cc} {\dot{\psi }}_{m} & =\Xi \left[\left(I_{3\times 3}-C_{m}^{s_{c}}C_{s_{r}}^{m}\right)\omega_{ns_{c}}^{s_{c}}+C_{m}^{s_{c}}C_{s_{r}}^{m}\varepsilon^{s_{r}}\right]+w_{m}\\ \delta {\dot{V}}^{n} & =C_{s_{c}}^{n}\left(I_{3\times 3}-C_{m}^{s_{c}}C_{s_{r}}^{m}\right)f_{is_{r}}^{s_{r}}-\left(2\omega_{ie}^{n\times }+\omega_{en}^{n\times }\right)\delta V^{n}+C_{s_{r}}^{n}\nabla^{s_{r}}+w_{v}\\ {\dot{\varepsilon }}^{s_{r}} & =0_{3\times 1}\\ {\dot{\nabla }}^{s_{r}} & =0_{3\times 1}\\ {\dot{\psi }}_{a} & =\eta_{a} \end{array}\right.$$

The modeling of observation relies on the matching scheme of RTA. In this paper, the measurable misalignment angles and velocity error are chosen as observations. That is:

(6) $$z=\left[\begin{array}{c} \Theta (C_{n}^{s_{c}}C_{m}^{n})\\ V_{se}^{n}-V_{me}^{n} \end{array}\right]$$

where Θ(·) represents the function that calculates the Euler angle from the attitude transformation matrix; $V_{se}^{n}$ and $V_{me}^{n}$ denote the velocity of SINS and MINS projected in their own body frame, respectively.

The observation model is given by:

(7) $$z_{k}=H_{k}x_{k}+v_{k}$$

where $v_{k}$ is the observation noise. The coefficient matrix $H_{k}$ is described as:

(8) $$H_{k}=\left[\begin{array}{ccccc} I_{3\times 3} & 0_{3\times 3} & 0_{3\times 3} & 0_{3\times 3} & 0_{3\times 3}\\ 0_{3\times 3} & I_{3\times 3} & 0_{3\times 3} & 0_{3\times 3} & 0_{3\times 3} \end{array}\right]$$

The system modeling of RTA implies that many factors could affect the precision of observations provided by MINS; for instance, the unreliable signal from the external aid of the device, the random time-delay effect, the coupling effect of the lever arm and deformation, etc. These unpredictable disturbances may induce uncertain statistics of observation noise, which motivates the investigation of the nonlinear filtering method to improve both the accuracy and robustness of RTA.

## 3. Stochastic Integration H${}_{\infty }$ Filter

In this section, the derivations of SIH${}_{\infty }$F are described in detail. First, the preliminaries of the H${}_{\infty }$ technique and EH${}_{\infty }$F are briefly represented. Then, the general form of DFH${}_{\infty }$F is given as the prerequisite of derivations. At last, the construction of SSRIR is introduced to evaluate the Gaussian weighted integrals in DFH${}_{\infty }$F, and SIH${}_{\infty }$F can be consequently achieved by combining the SSRIR with the framework of DFH${}_{\infty }$F.

### 3.1. Preliminaries of the H${}_{\infty }$ Technique

A representative nonlinear discrete-time system can be represented as follows:

(9) $$\left\{\begin{array}{c} x_{k}=f\left(x_{k-1}\right)+\omega_{k-1}\\ z_{k}=h\left(x_{k}\right)+v_{k}\\ y_{k}=L_{k}x_{k} \end{array}\right.$$

where $x_{k}\in R^{n}$ is the state vector; $z_{k}\in R^{m}$ is the observation vector; $y_{k}$ is the signal to be estimated; f(·) and h(·) are the process model and the observation model, respectively; $L_{k}$ is a known matrix, which will be replaced by the identity matrix for estimating the whole state vector in this study; the process noise $\omega_{k-1}$ and observation noise $v_{k}$ are assumed to be the signals with bounded energy, but unknown statistics, i.e., $\sum_{k=0}^{\infty }\omega_{k}^{T}\omega_{k}<\infty$, $\sum_{k=0}^{\infty }v_{k}^{T}v_{k}<\infty$.

Let $e_{k}=∥\hat{y_{k}}-y_{k}∥$ be the estimation error; the essential purpose of the H${}_{\infty }$ technique is to estimate $y_{k}$ to minimize $e_{k}$ under the worst case of initial estimation error, process noise and observation noise. For this purpose, the game theory approach gives a celebrated cost function, so as to map the disturbances and uncertainties to the estimation error. That is:

(10) $$J=\frac{\sum_{j=0}^{k}{∥\begin{array}{c} {\hat{y}}_{j}-x_{j} \end{array}∥}^{2}}{{∥\begin{array}{c} x_{0}-{\hat{x}}_{0} \end{array}∥}_{P_{0}^{-1}}^{2}+\sum_{j=0}^{k-1}{∥\begin{array}{c} \omega_{j} \end{array}∥}_{Q_{\omega ,j}^{-1}}^{2}+\sum_{j=0}^{k}{∥\begin{array}{c} v_{j} \end{array}∥}_{R_{v,j}^{-1}}^{2}}$$

where $P_{0}$ denotes the initial covariance of the state vector and $Q_{\omega ,j}$ and $R_{v,j}$ are the covariance of process noise and observation noise, respectively. The norm notation stands for ${∥\begin{array}{c} a \end{array}∥}_{B}^{2}=a^{T}Ba$.

The H${}_{\infty }$ filter sequentially estimates ${\hat{y}}_{j}$ to satisfy the following inequality:

(11) $$sup\;J<\gamma^{2}$$

where notation ‘sup’ stands for the supremum; γ represents the error attenuation parameter that is empirically set to a constant value or iteratively computed in real time [19,21].

### 3.2. Extended H${}_{\infty }$ Filter and Derivative-Free H${}_{\infty }$ Filter

As an intuitive extension of the linear H${}_{\infty }$ solution, the EH${}_{\infty }$F has been well studied to achieve suboptimal solutions for a nonlinear system with uncertainties [13,14]. The implementation of EH${}_{\infty }$F is represented as follows:

(12) $${\hat{x}}_{k|k-1}=f\left({\hat{x}}_{k-1|k-1}\right)$$

(13) $$P_{k|k-1}=F_{k-1}P_{k-1|k-1}F_{k-1}^{T}+Q_{\omega ,k-1}$$

(14) $$K_{k|k}=P_{k|k-1}H_{k}^{T}{\left(H_{k}P_{k|k-1}H_{k}^{T}+R_{v,k}\right)}^{-1}$$

(15) $${\hat{x}}_{k|k}={\hat{x}}_{k|k-1}+K_{k|k}\left[z_{k}-h\left({\hat{x}}_{k|k-1}\right)\right]$$

(16) $$R_{e,k}^{}=\left[\begin{array}{cc} R_{v,k}+H_{k}^{}P_{k|k-1}H_{k}^{T} & H_{k}^{}P_{k|k-1}\\ P_{k|k-1}H_{k}^{T} & P_{k|k-1}-\gamma^{2}I \end{array}\right]$$

(17) $$P_{k|k}=P_{k|k-1}-\left[\begin{array}{cc} P_{k|k-1}H_{k}^{T} & P_{k|k-1} \end{array}\right]R_{e,k}^{-1}\left[\begin{array}{c} H_{k}P_{k|k-1}\\ P_{k|k-1} \end{array}\right]$$

where ${\hat{x}}_{k|k-1}$ is the predicted mean with covariance $P_{k|k-1}$; ${\hat{x}}_{k|k}$ is the filtered mean with covariance $P_{k|k}$; $F_{k-1}$ and $H_{k}$ represent the Jacobian matrices of f(·) evaluated at the filtered mean ${\hat{x}}_{k-1|k-1}$ and h(·) evaluated at the predictive mean ${\hat{x}}_{k|k-1}$, respectively; $K_{k|k}$ is the gain matrix; $R_{e,k}$ is the auxiliary matrix.

Though the first-order Taylor expansion is utilized in EH${}_{\infty }$F to approximate the nonlinear model, the rough linearization method may degrade the estimation accuracy or even induce divergence in the presence of significant nonlinearity. Moreover, the computation of Jacobians is also an inevitable difficulty for the complicated model description. To overcome the limitations of EH${}_{\infty }$F, multiple DFH${}_{\infty }$Fs have been proposed on the basis of deterministic sampling quadrature methods. Without loss of generality, the general form of DFH${}_{\infty }$F is represented as follows.

Analogous to the nonlinear filter with the Gaussian assumption, the prediction step of DFH${}_{\infty }$F is given by:

(18) $${\hat{x}}_{k|k-1}=\int f\left(x_{k-1}\right)N\left\{x_{k-1};{\hat{x}}_{k-1|k-1},P_{k-1|k-1}\right\}\;dx_{k-1}$$

(19) $$P_{k|k-1}=\int \left[f\left(x_{k-1}\right)-{\hat{x}}_{k|k-1}\right]{\left[f\left(x_{k-1}\right)-{\hat{x}}_{k|k-1}\right]}^{T}N\left\{x_{k-1};{\hat{x}}_{k-1|k-1},P_{k-1|k-1}\right\}\;dx_{k-1}+Q_{\omega ,k-1}$$

where $N\{x_{k-1};{\hat{x}}_{k-1|k-1},P_{k-1|k-1}\}$ denotes the variable $x_{k-1}$ subject to the Gaussian probability distribution function with mean ${\hat{x}}_{k-1|k-1}$ and covariance $P_{k-1|k-1}$, and this notation is available for other equations.

Under the assumption that the integrated state vector needs to be estimated, the filtering step of DFH${}_{\infty }$F can be described as:

(20) $${\hat{x}}_{k|k}={\hat{x}}_{k|k-1}+K_{k|k}\left(z_{k}-{\hat{z}}_{k|k-1}\right)$$

(21) $$\begin{array}{cc} P_{k|k} & =P_{k|k-1}-\left[\begin{array}{cc} P_{xz,k|k-1} & P_{k|k-1} \end{array}\right]R_{e,k}^{-1}{\left[\begin{array}{cc} P_{xz,k|k-1} & P_{k|k-1} \end{array}\right]}^{T} \end{array}$$

where $K_{k|k}=P_{xz,k|k-1}{\left(P_{zz,k|k-1}+R_{v,k}\right)}^{-1}$ is the gain matrix; ${\hat{z}}_{k|k-1}$ is the predicted observation with covariance $P_{zz,k|k-1}$; $P_{xz,k|k-1}$ is the cross-covariance between the state vector and observation vector. Note that these three statistics of observation involve the computation of Gaussian weighted integrals, that is:

(22) $${\hat{z}}_{k|k-1}=\int h\left(x_{k}\right)N\left\{x_{k};{\hat{x}}_{k|k-1},P_{k|k-1}\right\}\;dx_{k}$$

(23) $$P_{zz,k|k-1}=\int \left[h\left(x_{k}\right)-{\hat{z}}_{k|k-1}\right]{\left[h\left(x_{k}\right)-{\hat{z}}_{k|k-1}\right]}^{T}N\left\{x_{k};{\hat{x}}_{k|k-1},P_{k|k-1}\right\}\;dx_{k}+R_{v,k}$$

(24) $$P_{xz,k|k-1}=\int \left(x_{k}-{\hat{x}}_{k|k-1}\right){\left[h\left(x_{k}\right)-{\hat{z}}_{k|k-1}\right]}^{T}N\left\{x_{k};{\hat{x}}_{k|k-1},P_{k|k-1}\right\}\;dx_{k}$$

The auxiliary matrix $R_{e,k}$ is given by:

(25) $$\begin{array}{c} R_{e,k}=\left[\begin{array}{cc} R_{v,k}+P_{zz,k|k-1} & P_{xz,k|k-1}^{T}\\ P_{xz,k|k-1} & P_{k|k-1}-\gamma^{2}I \end{array}\right] \end{array}$$

In contrast to the EH${}_{\infty }$F, the prediction step of DFH${}_{\infty }$F is explicitly replaced by the general Gaussian approximations, i.e., Equations (18) and (19). Furthermore, on the basis of the statistical linear error propagation method, the statistics related to Jacobian matrix $H_{k}$ in Equations (13), (16) and (17) are substituted by the following approximations [26]:

(26) $$P_{zz,k|k-1}=E\left[\left(z_{k}-{\hat{z}}_{k|k-1}\right){\left(z_{k}-{\hat{z}}_{k|k-1}\right)}^{T}\right]\approx H_{k}P_{k|k-1}H_{k}^{T}\;\left(k=1,2,\cdots \right)$$

(27) $$P_{xz,k|k-1}=E\left[\left(x_{k}-{\hat{x}}_{k|k-1}\right){\left(z_{k}-{\hat{z}}_{k|k-1}\right)}^{T}\right]\approx P_{k|k-1}H_{k}^{T}\;\left(k=1,2,\cdots \right)$$

### 3.3. Stochastic Spherical-Radial Integration Rule

It is worth noting that the general form of DFH${}_{\infty }$F requires the evaluation of Gaussian weighted integrals with the form $I\left(g\right)=\int g\left(x\right)N\left\{x;\hat{x},P\right\}\;dx$. In general, these nonlinear integrals cannot be analytically solved, and the numerical approximation method is required. Contrary to the previous deterministic sampling methods, in this paper, the SSRIR is employed for the evaluation of I(g). Note that two transformations must be implemented to convert the nonlinear integral expression to the standard form.

The first transformation relates to the integration variable, i.e., $x=\hat{x}+S_{x}u$, where $S_{x}$ is the lower triangular matrix obtained from the Cholesky decomposition of covariance P. That is:

(28) $$\begin{array}{cc} I(g) & =\int g\left(x\right)N\left\{x;\hat{x},P\right\}\;dx\\ & =\int g\left(\hat{x}+S_{x}u\right)N\left\{u;0_{n_{x}\times 1},I_{n_{x}}\right\}\;du \end{array}$$

where $n_{x}$ is the dimension of x.

The second transformation concerns the conversion of the integral variable to the radial-spherical coordinate system. Let $u=r\hat{u}$, where ${\hat{u}}^{T}\hat{u}=1$. The domain of definition for radius is r∈[0,∞). The integral can be rewritten as:

(29) $$\begin{array}{cc} I(g) & =\frac{1}{2}\int_{-\infty }^{\infty }\int_{∥\hat{u}{∥}_{2}=1}g\left(\hat{x}+S_{x}u\right){\left|r\right|}^{n_{x}-1}N\left\{r;0,1\right\}\;d\hat{u}dr \end{array}$$

In this stage, I(g) can be regarded as the combination of radial integration with the form $I_{r}\left(g_{r}\right)=\int_{-\infty }^{\infty }g_{r}\left(r\right){\left|r\right|}^{n_{x}-1}N\left\{r;0,1\right\}dr$ and spherical integration for the unit $n_{x}$-sphere with the form $I_{s}\left(g_{s}\right)=\int_{{∥\hat{u}∥}_{2}=1}g_{s}\left(\hat{u}\right)d\hat{u}$. The stochastic radial integration rule (SRIR) and stochastic spherical integration rule (SSIR) can be obtained from the following two Lemmas [25]:

**Lemma** **1.** *Assuming a set of weights ${\left\{\omega_{r,j}\right\}}_{j=0}^{m_{r}}$ are computed by the following equation:*

(30) $$\omega_{r,j}=\int_{-\infty }^{\infty }{\left|r\right|}^{n_{x}-1}N\left\{r;0,1\right\}\prod_{t=0,t\neq j}^{m_{r}}\frac{r^{2}-\rho_{t}^{2}}{\rho_{j}^{2}-\rho_{t}^{2}}\;dr,\;j=0,1,\cdots ,m_{r}$$

*where $\rho_{0}=0$, and the set of sample points ${\left\{\rho_{j}\right\}}_{j=1}^{m_{r}}$ are extracted from the joint PDF $p\left(\rho_{1},\cdots ,\rho_{m_{r}}\right)\propto \prod_{j=1}^{N_{r}}\rho_{j}^{n_{x}+1}N\left\{\rho_{j};0,1\right\}\prod_{t=1}^{j-1}{\left(\rho_{j}-\rho_{t}\right)}^{2}\left(\rho_{j}+\rho_{t}\right)$. Then, an unbiased $d_{r}=2m_{r}+1$ degree SRIR can be obtained for $I_{r}\left(g_{r}\right)$, that is:*

(31) $${\bar{I}}_{r}\left(g_{r}\right)\;\;\approx \frac{1}{2}\sum_{j=0}^{m_{r}}\omega_{r,j}\left[g_{r}\left(\rho_{j}\right)+g_{r}\left(-\rho_{j}\right)\right]$$

**Lemma** **2.** Let ${\bar{I}}_{s}\left(g_{s}\right)\;=\sum_{i=0}^{m_{s}}\omega_{s,i}g_{s}\left({\hat{u}}_{i}\right)$ be a deterministic $d_{s}$-degree spherical integration rule for $I_{s}\left(g_{s}\right)$, and Q is a uniformly chosen orthogonal matrix. Then, ${\bar{I}}_{Qs}\left(g_{s}\right)\;=\sum_{i=0}^{m_{s}}\omega_{s,i}g_{s}\left(Q{\hat{u}}_{i}\right)$ is an unbiased $d_{s}$-degree SSIR for $I_{s}\left(g_{s}\right)$.

Note, the selection of weights ${\left\{\omega_{s,i}\right\}}_{i=0}^{m_{s}}$ and sample points ${\left\{{\hat{u}}_{i}\right\}}_{i=0}^{m_{s}}$ is not unique; various deterministic spherical integration rules are given in [25].

In this paper, a three-degree SRIR and three-degree SSIR are adopted. Especially, the three-degree spherical integration rule in Lemma 2 is given by:

(32) $${\bar{I}}_{3,s}\left(g_{s}\right)\;=\frac{∥U_{n_{x}}∥}{2n_{x}}\sum_{i=1}^{n_{x}}\left(g_{s}\left(-e_{i}\right)+g_{s}\left(e_{i}\right)\right)$$

where $U_{n_{x}}$ is the surface of the unit $n_{x}$-sphere; $∥U_{n_{x}}∥=2\pi^{n_{x}/2}/\Gamma \left(n_{x}/2\right)$ is the surface content of $U_{n_{x}}$; $e_{i}$ denotes the unit vector with “1” in the *i*-th element and “0” in other positions.

The SSRIR can be obtained by constructing a product of a $d_{r}$-degree SRIR and a $d_{s}$-degree SSIR, that is:

(33) $$\begin{array}{cc} {\bar{I}}_{}^{\left(d_{r},d_{s}\right)}\left(g\right) & =\frac{1}{4}\sum_{j=0}^{m_{r}}\omega_{r,j}\sum_{i=0}^{m_{s}}\omega_{s,i}\left[g\left(\hat{x}\pm \rho_{j}S_{x}Q{\hat{u}}_{i}\right)+\bar{g}\left(\hat{x}\pm \rho_{j}S_{x}Q{\hat{u}}_{i}\right)\right]\\ & =\sum_{k=1}^{N^{SR}}\omega_{k}^{SR}g\left(\chi_{k}\right) \end{array}$$

where $N^{SR}$ denotes the total number of the sample points; $\chi_{k}=\hat{x}\pm \rho_{j}S_{x}Q{\hat{u}}_{i}$ is the sample point with weight $\omega_{k}^{SR}=\omega_{s,i}^{}\omega_{r,j}^{}/4\;$.

The approximation of $I\left(g\right)$ based on SSRIR with *N* iterations can be obtained as:

(34) $$I_{N}^{\left(d_{r},d_{s}\right)}\left(g\right)=\frac{1}{N}\sum_{l=1}^{N}{\bar{I}}_{l}^{\left(d_{r},d_{s}\right)}\left(g\right)$$

where ${\bar{I}}_{l}^{\left(d_{r},d_{s}\right)}\left(g\right)$ denotes the *l*-th ${\bar{I}}_{}^{\left(d_{r},d_{s}\right)}\left(g\right)$.

In theory, the statistics of function $g\left(x\right)$, including the mean, covariance and cross-covariance, should be separately approximated by SSRIR. However, in consideration of the computational cost, these statistics are commonly approximated in a single run of SSRIR [22]. For completeness, the detailed implementation of SSRIR with constant iterations is represented as follows:

1. Initialize the number of iterations l=1. Define the maximum number of iterations as $N_{its}$. Set the initial mean value $\mu_{g}=0_{n_{g}\times 1}$, initial covariance $P_{gg}=0_{n_{g}\times n_{g}}$ and initial cross-covariance $P_{xg}=0_{n_{g}\times n_{g}}$; where $n_{g}$ is the dimension of g(x).
2. While $1\leq l\leq N_{its}$, use Equation (33) to generate the SSRIR-based sample points $\chi ={\left\{\chi_{l\left(k\right)}\right\}}_{k=1}^{N_{}^{SR}}$ and corresponding weights $\omega_{}^{SR}={\left\{\omega_{l\left(k\right)}^{SR}\right\}}_{k=1}^{N_{}^{SR}}$ at the *l*-th iteration.
3. Calculate the statistics of g(x) at the current iteration:
  (35) $$\mu_{g}=\frac{1}{N}\sum_{l=1}^{N}\sum_{k=1}^{N_{}^{SR}}\omega_{l\left(k\right)}^{SR}g\left(\chi_{l\left(k\right)}\right)$$
  (36) $$P_{gg}=\frac{1}{N}\sum_{l=1}^{N}\sum_{k=1}^{N_{}^{SR}}\omega_{l\left(k\right)}^{SR}g\left(\chi_{l\left(k\right)}\right)g{\left(\chi_{l\left(k\right)}\right)}^{T}-\mu_{g}{\left(\mu_{g}\right)}^{T}$$
  (37) $$P_{xg}=\frac{1}{N}\sum_{l=1}^{N}\sum_{k=1}^{N_{}^{SR}}\omega_{l\left(k\right)}^{SR}\chi_{l\left(k\right)}g{\left(\chi_{l\left(k\right)}\right)}^{T}-\mu_{x}{\left(\mu_{g}\right)}^{T}$$
4. Set l=l+1, and shift to Step 2.

For simplicity, the implementation of SSRIR can be compactly denoted as:

(38) $$\left[\mu_{g},P_{gg},P_{xg}\right]=\mathrm{SSRIR}\left[g\left(\cdot \right),\hat{x},P\right]$$

If the process model in (9) is nonlinear, Equations (18) and (19) can be substituted with:

(39) $$\left[{\hat{x}}_{k|k-1},\left(P_{k|k-1}-Q_{\omega ,k-1}\right)\right]=\mathrm{SSRIR}\left[f\left(\cdot \right),{\hat{x}}_{k-1|k-1},P_{k-1|k-1}\right]$$

Analogously, if the observation model in (9) is nonlinear, Equations (22)–(24) can be substituted with:

(40) $$\left[{\hat{z}}_{k|k-1},\left(P_{zz,k|k-1}-R_{v,k}\right),P_{xz,k|k-1}\right]=\mathrm{SSRIR}\left[h\left(\cdot \right),{\hat{x}}_{k|k-1}^{},P_{k|k-1}\right]$$

**Figure 3:**
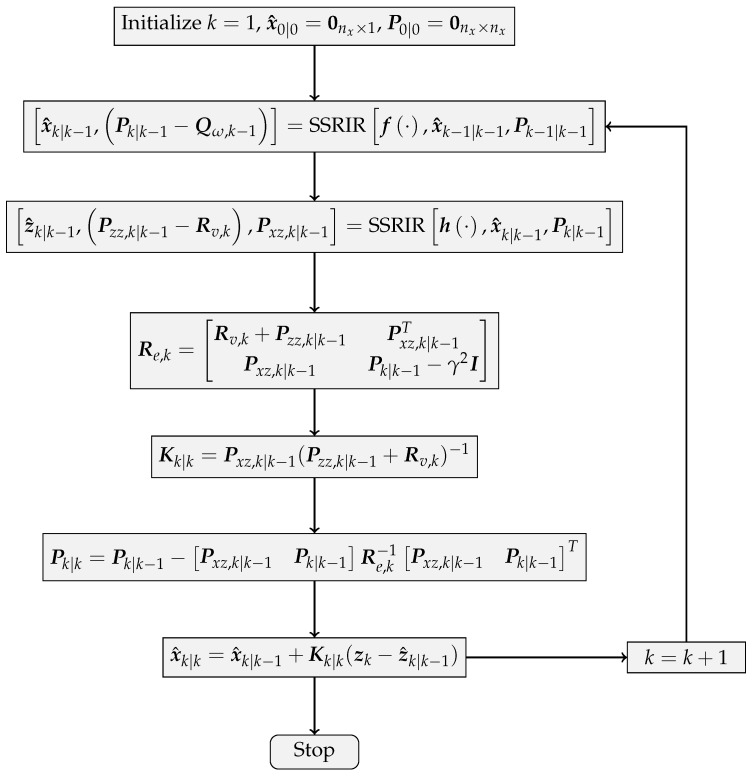
Flowchart of the SIH${}_{\infty }$F algorithm.

Eventually, Equations (20), (21), (25), (39) and (40) are composed of the integrated SIH${}_{\infty }$F method. The flowchart of the SIH${}_{\infty }$F algorithm is shown in Figure 3.

Figure 3 implies that the SSRIR is incorporated with the framework of DFH${}_{\infty }$F so as to approximate the nonlinear function in the model description. Compared with the traditional SIF, the SIH${}_{\infty }$F introduces the cost function described in Equation (10) and aims to estimate the state under the worst initial error and model uncertainty.

## 4. Numerical Simulation

Numerical simulation is an essential approach to analyze the performance of the proposed method. As a prerequisite, a flight trajectory is designed to generate the simulated data of MINS and SINS. Then, the simulated data of the two INSs are processed by different filtering algorithms for comparison.

### 4.1. Designs of the Numerical Simulation

**Figure 4:**
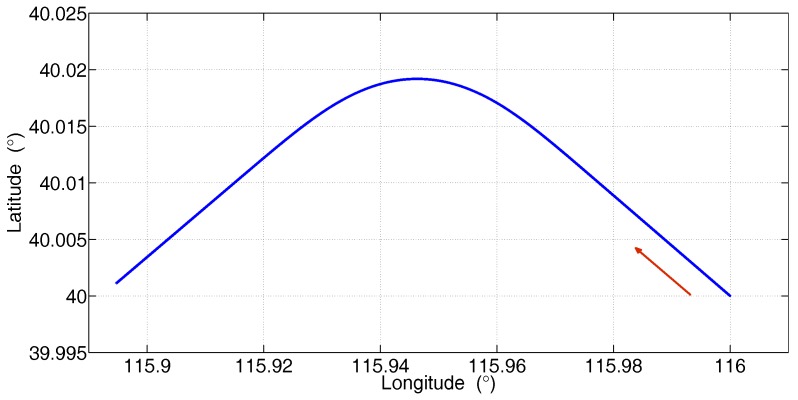
Trajectory of the simulation.

In this subsection, a typical flight trajectory with a turning maneuver is designed, as shown in Figure 4.

Firstly, the aircraft flies 20 s with a constant velocity, then turns anticlockwise $60^{\circ }$ in 60 s and continually flies 20 s with a constant velocity. Note that the roll angle and yaw angle of the aircraft are changing simultaneously during the turning maneuver. The specifications of the simulated flight trajectory are given in Table 1.

The initial actual physical misalignment angles are set to $\psi_{a}=\left[5^{\circ };\;5^{\circ };\;80^{\circ }\right]$ with covariance $Q_{a}=diag\left\{{\left(0.02^{\circ }/h\right)}^{2};\;{\left(0.02^{\circ }/h\right)}^{2};\;{\left(0.02^{\circ }/h\right)}^{2}\right\}$. The errors of inertial instruments in SINS are listed as follows: the constant drift of the gyroscope is $5^{\circ }/h$, and the random walk of the gyroscope is $1^{\circ }/\sqrt{h}$; the constant bias of accelerator is 0.2 mg, and the random walk of accelerator is 0.02 m/s/$\sqrt{h}$. The attitude precision and velocity precision of MINS are $0.1^{\circ }$ and 0.02 m/s, respectively. The observation noise with uncertain statistics is achieved by injecting non-Gaussian noise in the simulated observations. The disturbing noise for attitude observation is assumed to satisfy the uniform distribution within $\left[-0.05^{\circ }\;0.05^{\circ }\right]$. Similarly, the disturbing noise for velocity observation is assumed to satisfy the uniform distribution within [−0.01 m/s 0.01 m/s].

### 4.2. Results and Discussion

**Figure 5:**
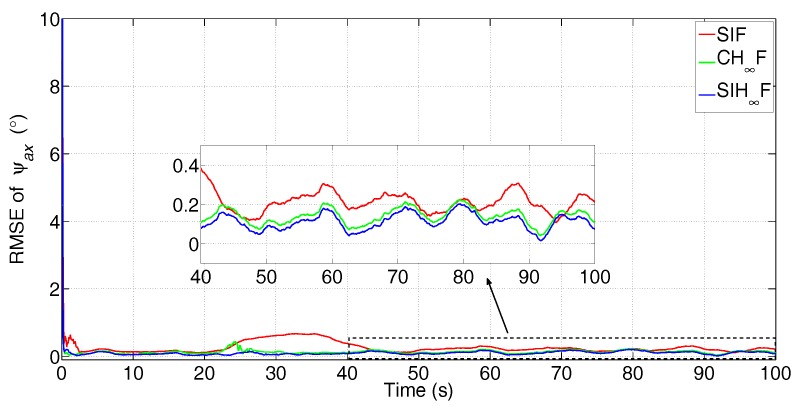
RMSE of $\psi_{ax}$ (the subfigure is the magnification of the estimation result during 40 s–100 s).

**Figure 6:**
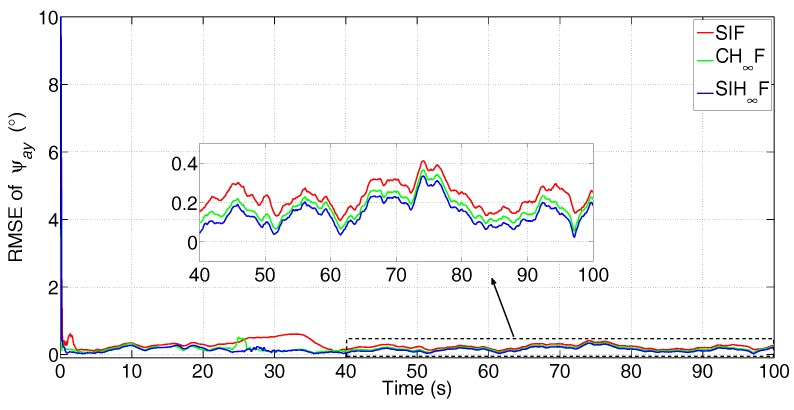
RMSE of $\psi_{ay}$ (the subfigure is the magnification of the estimation result during 40 s–100 s).

**Figure 7:**
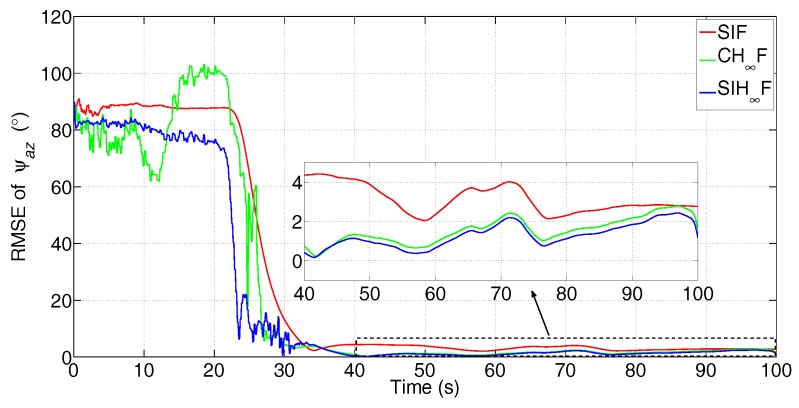
RMSE of $\psi_{az}$ (the subfigure is the magnification of the estimation result during 40 s–100 s).

The simulated data are processed by SIF, CH${}_{\infty }$F and SIH${}_{\infty }$F, respectively. The error attenuation parameter for CH${}_{\infty }$F and SIH${}_{\infty }$F is set to γ=0.08. The number of iterations of SIF and SIH${}_{\infty }$F is $N_{its}=20$. These methods were independently performed with 50 Monte Carlo simulations, and the root-mean-square error (RMSE) of the three misalignment angles was utilized to evaluate the performance of different filtering algorithms with statistical significance. The estimation results are shown in Figure 5, Figure 6 and Figure 7.

From Figure 5 and Figure 6, it can be seen that all three methods have a quick convergence performance for the estimation of horizontal misalignment angles, i.e., $\psi_{ax}$ and $\psi_{ay}$. Meanwhile, the estimation results of $\psi_{az}$ are partially estimated at the beginning of RTA and quickly converged to true values while the turning maneuver of aircraft is carried out.

Among these methods, SIF gives poor performance in terms of all three misalignment angles, as it requires accurate statistics of the noise. However, the existence of non-Gaussian noise in the observation degrades the performance of SIF. Especially, Figure 5 and Figure 6 show that SIF cannot suppress the negative effect of uncertainties at the beginning of the turning maneuver (20 s). In contrast, CH${}_{\infty }$F has better performance than SIF, since the combination of the H${}_{\infty }$ technique and cubature integration rule simultaneously suppressed the negative effect of uncertainties and nonlinearities. Even so, the estimation given by CH${}_{\infty }$F fluctuates wildly for the estimation of $\psi_{az}$, as is shown in Figure 7. This phenomenon demonstrates that the local validity of the cubature integration rule cannot guarantee the consistency of the estimation error with the existence of uncertainty and nonlinearity. The SIH${}_{\infty }$F provides the highest estimation accuracy compared with other methods. Besides, the estimations of SIH${}_{\infty }$F are obviously smoother than CH${}_{\infty }$F. This demonstrates that the SSRIR can approximate the nonlinear function more accurately than the cubature integration rule, and the boundedness of estimation errors can be guaranteed. As a result, the performance of SIH${}_{\infty }$F outperforms the CH${}_{\infty }$F and SIF in the statistical sense.

The statistics of RMSE in the last 20 s are used to further evaluate the accuracy and stability of different filtering algorithms. The mean values (in the time-averaged sense) and standard deviation (STD) values of 50 dependent simulations are given in Table 2.

From Table 2, it is obvious that the results given by SIH${}_{\infty }$F are superior to other methods in terms of both mean and STD values, since it possesses a high ability to address the coexistence of nonlinearity and uncertainty.

**Figure 8:**
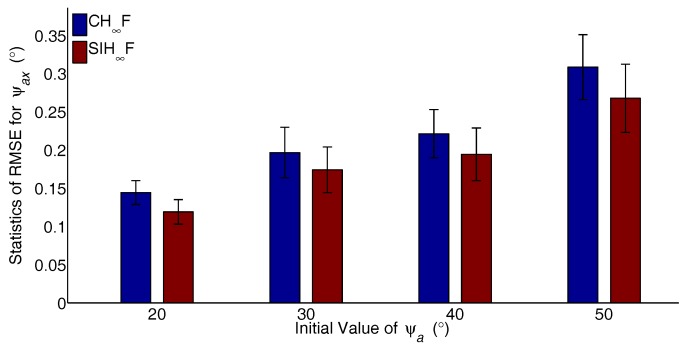
Statistics of RMSE for $\psi_{ax}$.

**Figure 9:**
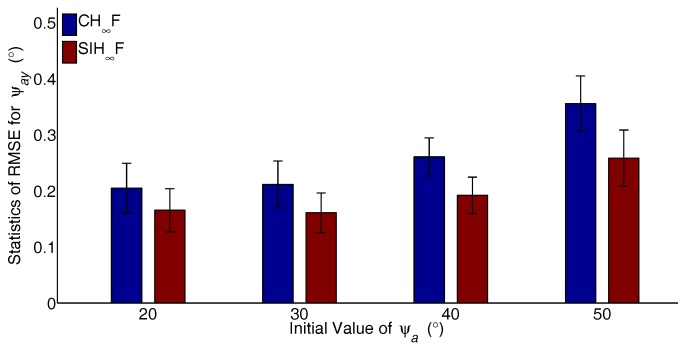
Statistics of RMSE for $\psi_{ay}$.

**Figure 10:**
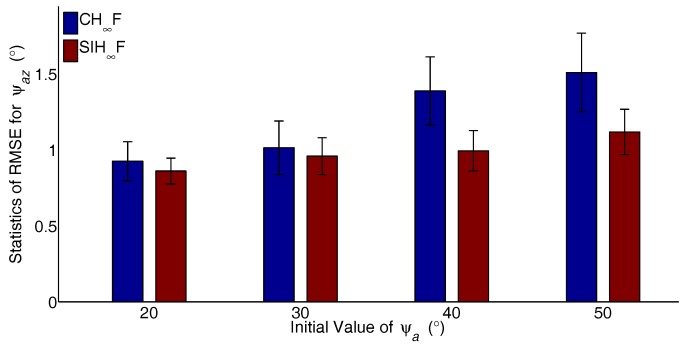
Statistics of RMSE for $\psi_{az}$.

The major advantage of SIH${}_{\infty }$F is the ability to address the estimation problem with significant nonlinearity. To verify the performance of the proposed method, different cases with increasing degrees of nonlinearity are designed. The initial values of $\psi_{ai},\left(i=x,y,z\right)$ are set to $20^{\circ }$–$50^{\circ }$ with $10^{\circ }$ intervals, and the RTA based on CH${}_{\infty }$F and SIH${}_{\infty }$F were independently carried out 50 times for each case. The statistics of RMSE, including the mean and STD for the last 20 s, are utilized to evaluate the performance of the filters. The resulting statistics of the RMSE are represented in Figure 8, Figure 9 and Figure 10.

As shown in Figure 8, Figure 9 and Figure 10, the estimation errors of CH${}_{\infty }$F obviously increase with the growth of initial actual misalignment angles. The result implies that the cubature integration rule based on the deterministic sampling strategy deteriorates the approximation accuracy of Gaussian weighted nonlinear integrals. In contrast, SIH${}_{\infty }$F performs better than CH${}_{\infty }$F, since the SSRIR provides asymptotically unbiased approximations for Gaussian weighted nonlinear integrals. In other words, SIH${}_{\infty }$F alleviates the impact of significant nonlinearity, and the results demonstrate the validity of the proposed method.

## 5. Van Test

**Figure 11:**
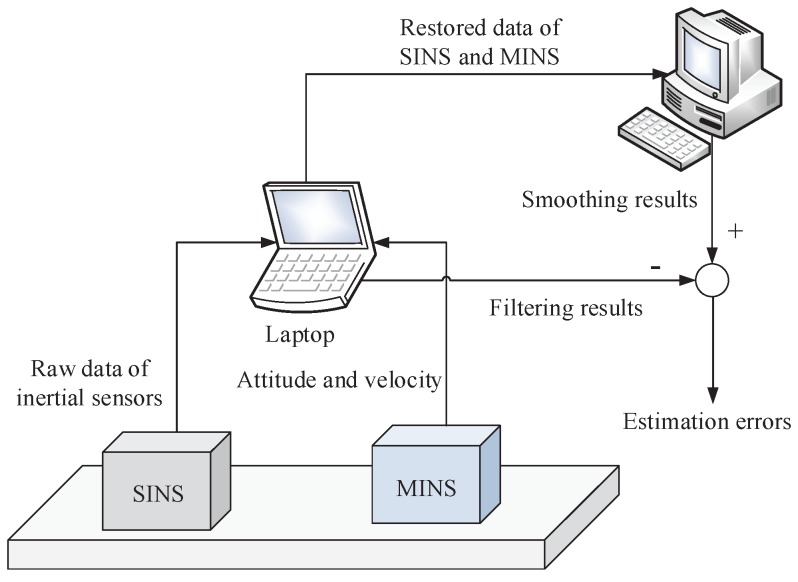
The structure diagram of the van test.

To further verify the performance of the investigated method, a van test was carried out. In this van test, the RTA is implemented while MINS and SINS are operated on a moving base. SINS outputs the raw data of inertial sensors, including the angular velocity and specific force, while MINS provides the observation data, i.e., the attitude and velocity. By using different filtering algorithms, the RTA procedure provides the estimation results of misalignment angles in real time. Besides, an accuracy assessment procedure is also necessitated to evaluate the performance of different filters. The structure diagram of the van test is displayed in Figure 11.

### 5.1. Specifications of the Van Test

**Figure 12:**
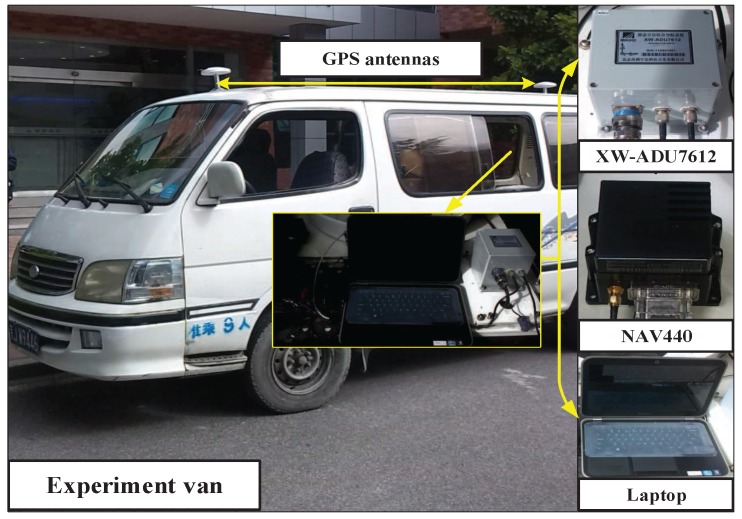
Configurations of the van test.

The test system contains a MEMS-based INS (NAV4400), an attitude and heading reference system (XW-ADU7612) and a laptop. The NAV440 is utilized as SINS to provide raw data of inertial sensors, and its dominating errors are listed as follows: the in-run stability of the gyroscope is $10^{\circ }/h$; the random walk of the gyroscope is $4.5^{\circ }/\sqrt{h}$; the in-run stability of the accelerometer is 1 mg; the random walk of the accelerometer is 1 m/s/$\sqrt{h}$. The XW-ADU7612 is taken as MINS. It contains an inertial measurement unit, whose accumulative errors are corrected by a GPS module with two external antennas. The attitude precision and velocity precision of MINS are $0.2^{\circ }$ and 0.02 m/s, respectively. The two INSs were bolted on a steel plate, and the GPS antennas were installed on top of the van. The update rates of MINS and SINS are 10 Hz and 100 Hz, respectively. The laptop is used for restoring the output data of the two INSs. The configurations of the van test are shown Figure 12.

**Figure 13:**
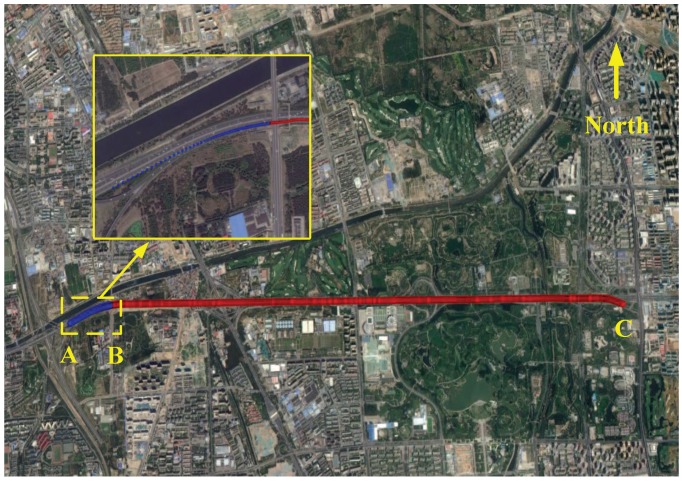
Trajectory of the van test.

The van test was performed in the northern fifth ring road of Beijing city, China. The trajectory of the van test is shown in Figure 13, in which the blue line denotes the trajectory for the implementation of RTA. The RTA was initiated at Point A and ended at Point B, and the execution time was 120 s.

**Figure 14:**
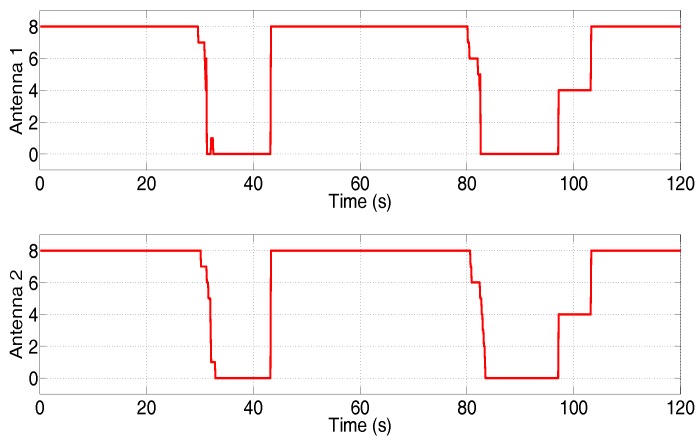
Number of captured satellites by the GPS antennas.

The GPS signal of MINS was intentionally interrupted two times during the test, that is the observations can be deduced to contain non-Gaussian noise. The number of captured satellites by the GPS antennas is shown in Figure 14.

### 5.2. Accuracy Assessment, Test Results and Discussions

For the purpose of evaluating the estimation accuracy of different methods, high precision reference misalignment angles are required. However, the benchmark of misalignment angles cannot be directly obtained in real time, and thus, the off-line accuracy assessment approach is necessitated. In this test, the relative attitude between MINS and SINS is invariable since they were rigidly bolted on the steel plate, i.e., $\psi_{a}$ can be considered as a constant vector. Therefore, the CKF-based fixed-point smoother was employed in the accuracy assessment procedure, on account of the nonlinear characteristic of the model description [27,28]. As is shown in Figure 13, the accuracy assessment procedure was initiated at Point B and ended at Point C (700 s), where the GPS functioned well and the Gaussian assumption for the observation noise is accredited. The smoothing estimations of misalignment angles are ${\bar{\psi }}_{ax}=0.18^{\circ }$, ${\bar{\psi }}_{ay}=0.9^{\circ }$ and ${\bar{\psi }}_{az}=-90.5^{\circ }$.

**Figure 15:**
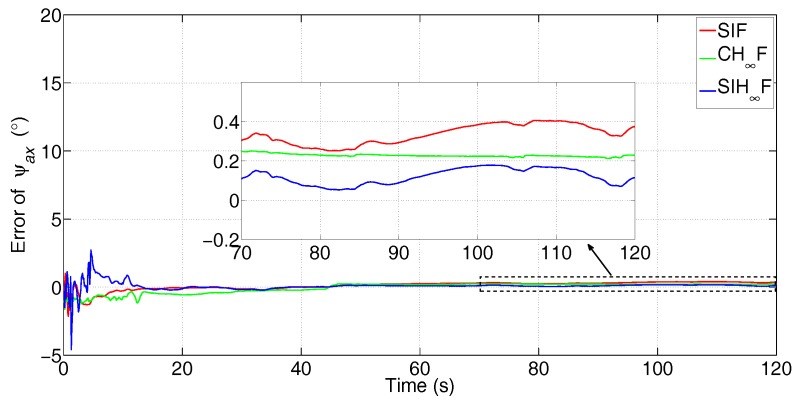
Error of $\psi_{ax}$ (the subfigure is the magnification of the estimation result during 70 s–120 s).

**Figure 16:**
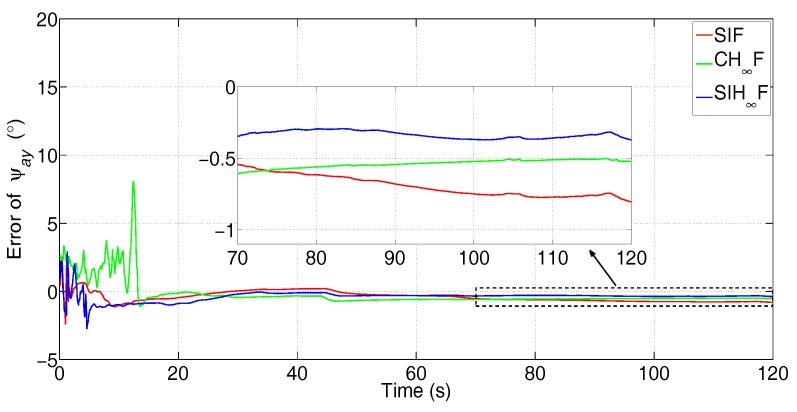
Error of $\psi_{ay}$ (the subfigure is the magnification of the estimation result during 70 s–120 s).

**Figure 17:**
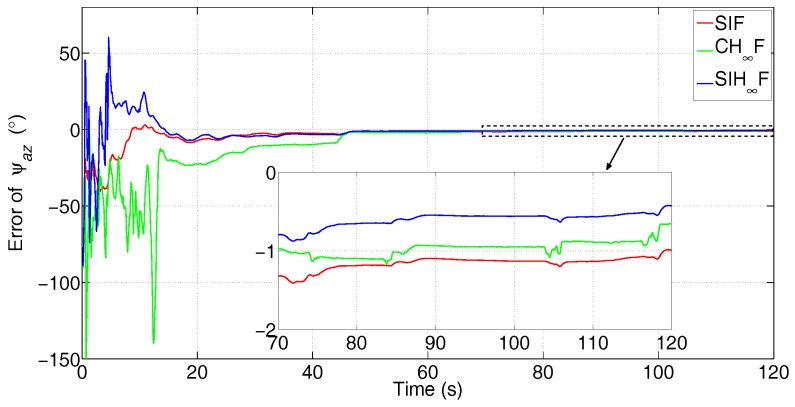
Error of $\psi_{az}$ (the subfigure is the magnification of the estimation result during 70 s–120 s).

In this test, the comparison between SIF, CH${}_{\infty }$F and SIH${}_{\infty }$F was executed. The error attenuation parameter for CH${}_{\infty }$F and SIH${}_{\infty }$F was set to γ=0.6. The the number of iterations of SIF and SIH${}_{\infty }$F was $N_{its}=20$. The estimation errors of the three methods are represented in Figure 15, Figure 16 and Figure 17.

Figure 15, Figure 16 and Figure 17 clearly demonstrate that the estimation accuracy of SIH${}_{\infty }$F outperforms SIF and CH${}_{\infty }$F in terms of all three misalignment angles. The oscillating amplitude of estimations given by CH${}_{\infty }$F is larger than that of SIH${}_{\infty }$F, since the approximation accuracy of the cubature integration rule is lower than that of SSRIR. The SIF cannot attenuate the influence caused by uncertain statistics of observation noise and, thus, gives the lowest estimation accuracy. After 120 s, the estimate errors of the three methods are shown in Table 3.

From Table 3, one can notice that the estimation precision of SIH${}_{\infty }$F is higher than SIF and CH${}_{\infty }$F, in which the precision of $\psi_{ax}$ increases by 69.5% and 49.9%, respectively, the precision of $\psi_{ay}$ increases by 53.7% and 28.8%, respectively, and the precision of $\psi_{az}$ increases by 56.9% and 34.5%, respectively.

## 6. Conclusions

The coexistence of large initial attitude errors and uncertain statistics of observation noise severely affects the accuracy of RTA. The major contribution of this paper is to develop a SIH${}_{\infty }$F for the purpose of improving the performance of RTA with considerations of accuracy and robustness. For the first time, the SSRIR is combined with the framework of DFH${}_{\infty }$F, and the resulting SIH${}_{\infty }$F can theoretically provide asymptotically unbiased estimation for a nonlinear system with high robustness. The numerical simulation and van test were carried out to compare the performance of the SIH${}_{\infty }$F, CH${}_{\infty }$F and traditional SIF. The results show that the newly-proposed method has the highest precision of the estimation results and robustness to the unknown statistics of observation noise. Promoted by its advantages, SIH${}_{\infty }$F can be considered as a competitive candidate method for the practical application of RTA.

## Figures and Tables

**Table 1:** The specifications of simulated flight trajectory.

Initial Attitude		Initial Velocity		Initial Position	
Yaw	60${}^{\circ }$	East	−86.6 m/s	Latitude	116${}^{\circ }$ N
Pitch	0${}^{\circ }$	North	50 m/s	Longitude	$40^{\circ }$ E
Roll	0${}^{\circ }$	Up	0 m/s	Height	1000 m

**Table 2:** Statistics of RMSE for 50 Monte Carlo simulations (${}^{\circ }$).

	Statistics	SIF	CH${}_{\infty }$F	SIH${}_{\infty }$F
$\psi_{ax}$	Mean	0.2101	0.1379	0.1122
	STD	0.0481	0.0402	0.0397
$\psi_{ay}$	Mean	0.2034	0.1489	0.1167
	STD	0.0468	0.0421	0.0411
$\psi_{az}$	Mean	2.7150	2.1596	1.8213
	STD	0.1460	0.4266	0.4134

**Table 3:** Estimation error of the van test (${}^{\circ }$).

	SIF	CH${}_{\infty }$F	SIH${}_{\infty }$F
$\psi_{ax}$	0.3750	0.2284	0.1145
$\psi_{ay}$	−0.8059	−0.5242	−0.3731
$\psi_{az}$	−0.9873	−0.6494	−0.4253
